# Exploring the Weathering and Accelerated Environmental Aging of Wave-Transparent Reinforced Composites

**DOI:** 10.3390/polym17030357

**Published:** 2025-01-28

**Authors:** Imran Haider, Muhammad Ali Khan, Shahid Aziz, Syed Husain Imran Jaffery, Muhammad Iftikhar Faraz, Iftikhar Hussain Gul, Dong-Won Jung, Taoufik Saidani, Walid M. Shewakh

**Affiliations:** 1 Thermal Transport Laboratory, Department of Materials Engineering, School of Chemical & Materials Engineering (SCME), National University of Sciences & Technology (NUST), Islamabad 44000, Pakistan; 2 School of Mechanical & Manufacturing Engineering (SMME), National University of Sciences & Technology (NUST), Islamabad 44000, Pakistan; mak.ceme@ceme.nust.edu.pk; 3 Department of Mechanical Engineering, College of Electrical and Mechanical Engineering (CEME), National University of Sciences and Technology (NUST), Islamabad 44000, Pakistan; 4 Department of Mechanical Engineering, Jeju National University, 102 Jejudaehak-ro, Jeju-si 63243, Republic of Korea; 5 Institute of Basic Sciences, Jeju National University, 102 Jejudaehak-ro, Jeju-si 63243, Republic of Korea; 6 Department of Mechanical Engineering, College of Engineering, Faculty of Computing, Engineering and the Built Environment Birmingham City University, Birmingham B4 7XG, UK; 7 Department of Mechanical Engineering, College of Engineering, King Faisal University, Al-Ahsa 31982, Saudi Arabia; 8 Faculty of Applied Energy System, Major of Mechanical Engineering, Jeju National University, 102 Jejudaehak-ro, Jeju-si 63243, Republic of Korea; 9 Center for Scientific Research and Entrepreneurship, Northern Border University, Arar 73213, Saudi Arabia; 10 Department of Industrial Engineering, College of Engineering and Computer Sciences, Jazan University, Jazan 82817, Saudi Arabia; 11 Mechanical Production Department, Faculty of Technology and Education, Beni-Suef University, P.O. Box 62521, Beni-Suef 62511, Egypt

**Keywords:** fiber composites, dielectric properties, FTIR spectra, microscale characterization

## Abstract

Approaches to retain or improve wave-transparent composite properties received ongoing attention. Silica glass fiber composites are being utilized in wave transparency applications owing to their excellent dielectric properties. During operational service life, they are exposed to ambient and harsh environments, which degrade their performance and properties. The objective is to evaluate the progressive degradation of silica fiber wave-transparent composite material’s properties and overall performance. Silica fiber/epoxy wave-transparent composites (SFWCs) were fabricated by stacking high-silica glass cloth (HSG) plies via multi-layer compression and curing at 150 °C (14 hrs) and were investigated upon one-year real-time weathering and 20-year accelerated aging (Hallberg peck model). The morphology of one-year-aged SFWC composite was found to be better than that of 20-year-aged SFWC, where relatively weakened interfacial bonding and composite structure were observed. One year weathering the dielectric constant (ε_r_) was increased to 4.34%, and dielectric loss (δ) was found to be 5.6%, whereas upon accelerated conditions (equivalent to 20 yrs of ambient conditions), ε_r_ was significantly raised 30.63% from its original value (3.2), and δ was increased 22.8% (0.035). In the 20-year aged SFWC composite, the maximum absorbed moisture was 3.1%. Tensile strength dropped from 147.8 MPa to 136.48 MPa, and compressive strength from 388.54 MPa to 374.41 MPa. Upon aging (from 1 year of weathering to 20 years of accelerated aging), SFWC composite properties and functional performance were lowered but remained reasonable. SFWC properties, as revealed by microscale characterization, can contribute to the determination of the impact of deterioration and useful service life in respective microelectronics wave transparency applications.

## 1. Introduction

**Figure 1:**
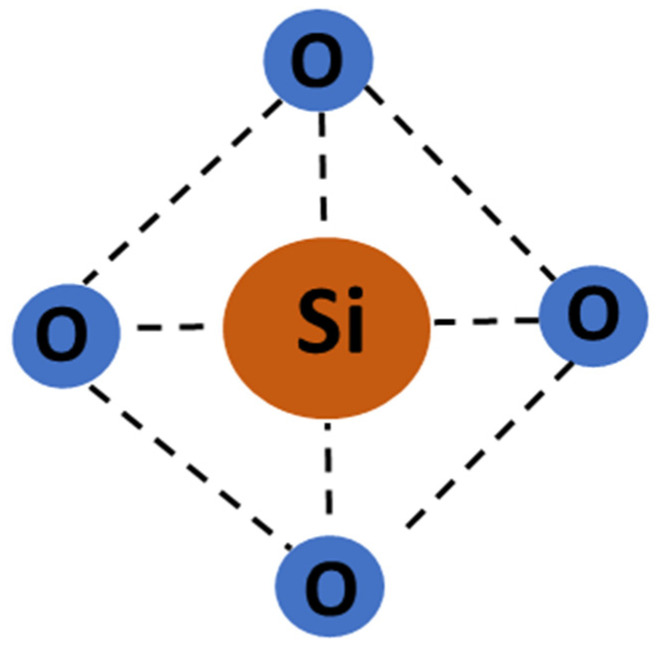
Silica fiber structure.

The transmission of electromagnetic waves and protection of communication equipment is critical in high-speed communication applications [1]. When integrated, the efficient performance of communication devices, modules, and circuit boards depend on the complete hardware, which includes antennas that transmit and receive signals [2]. For least interference in signal transmission [3] and environmental protection, these need to be guarded by wave-transparent material [2,4]. In this role, ceramics and fiber-reinforced composites are promising in protection from environmental conditions without compromising their wave transparency [5]. Glass fibers (GFs) are high-performance reinforcements to meet the functional requirements in wave transparency applications [6] due to their insulating nature [7]. GFs exhibit heat resistance, anti-corrosive properties, good mechanical stability, and long-term durability in various environmental conditions [8]. They (GFs) are amorphous, obtained from non-crystalline material [9] with a short-range network structure. Nature comprises a majority of the silica network (Figure 1), containing traces of calcium, sodium, boron, aluminum, and iron [10].

**Figure 2:**
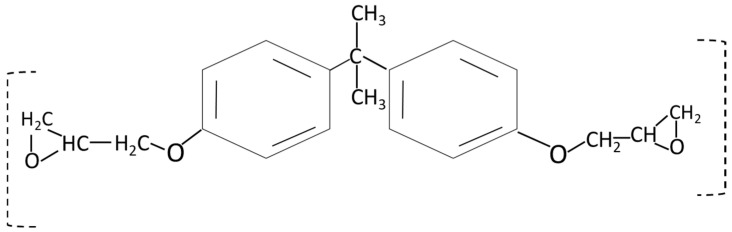
Chemical structure of the epoxy resin.

Simulating the environmental conditions can predict the earlier failure [11] or progressive degradation of composites [12]. Studying simulated conditions experimentally comes up with better output but is concerned with their deviation from real-time results [13]. This study presents the far environmental effects (weathering and accelerated environmental conditions) on the electrical and mechanical thermal properties of the SFWC composite. Long-term performance can determine their ability to withstand various environmental stressors without compromising their performance, physical, mechanical, and thermal integrity [14]. The durability of wave-transparent composites using cost-effective materials can lead to sustainability [15]. Epoxy resin is commonly used as resin in reinforced composites where toughness and durability are desired [16]. The low dielectric constant of epoxy resins makes them a suitable matrix for manufacturing low dielectric composites [17]. Figure 2 shows the basic chemical structure of the Bisphenol-A epoxy resin.

The tendency of wave-transparent composite to suffer deterioration upon environmental aging must not be underestimated but needs to be evaluated [18]. The determination of accurate weathering impact through real-time or accelerated conditions has been a never-ending attempt [3]. However, the motive is to evaluate the progressive degradation of wave-transparent composite material’s property and overall performance [19]. From an application point of view, the combined variation of SFWC (properties and functional performance) over the extended period is significant [20], but it is limitedly reported [21]. Considering this research gap, the present work investigates the impact of weathering vs. accelerated aging on the properties of silica fiber/epoxy wave-transparent composites (SFWCs). The SFWC were subjected to environmental exposure upon (i) one year weathering and (ii) accelerated condition. Among the aging conditions, the effect of temperature and relative humidity (% RH) on the morphology, dielectric, mechanical, and thermal structural properties; moisture absorption; and radome performance were determined. The objective of this study is to determine why and how much the high-silica glass fiber (HSG) composite properties degrade in real time due to accelerated weathering or harsh environmental conditions.

## 2. Materials and Methods

### 2.1. Composite Fabrication

Silica fiber-reinforced wave-transparent composites (SFWCs) were prepared by high-silica glass cloth (HSG, ρ = 2.2 g/cc, ε_r_ = 3.78, δ = 0.002, tensile modulus = 1.75 GPa, and softening point = 1600 °C) obtained from BET LHR, Pakistan. Adhesive epoxy resin (REH-100, RESICHEM KHI, Karachi, Pakistan) contained ρ = 1.25, ε_r_ = 3.5~4.1, δ = 0.02~0.03, and epoxy content = 186–200. HSG cloth was cut into plies and then died for 1 hr. at 140 C in a drying oven to remove the sizing. The matrix was prepared by mixing epoxy resin and hardener matrix in a ratio of 2.5:1 (epoxy).

**Figure 3:**
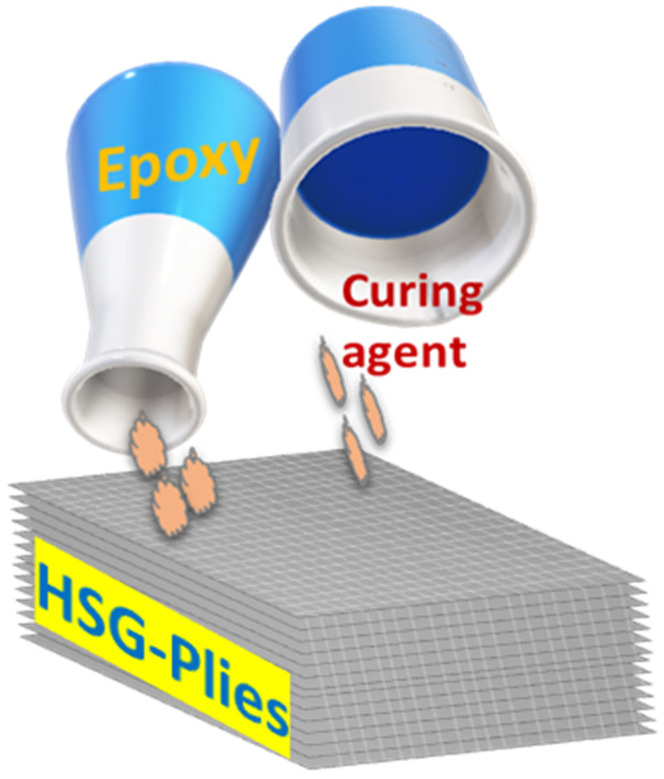
Pouring of resin on the silica fabric.

**Figure 4:**
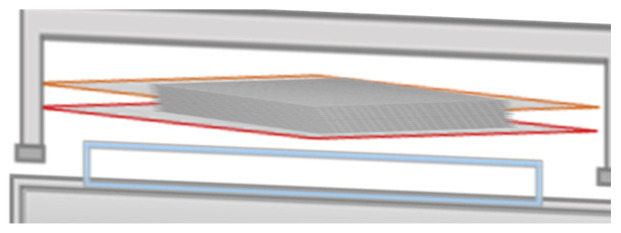
Arrangement of laminates in die-mold.

The solution was gently mixed (5 min) and diluted with methanol. The lab environmental conditions (45% RH and 26.5 °C), resin solution was applied on each HSG cloth ply and then stacked layer by layer as shown in Figure 3. The stacked HSG cloth laminate (one hundred plies) was placed in the stainless-steel die mold as shown in Figure 4, pressed at 50 bar and cured at 150 °C for 10 hrs, and post-cured for 4 hrs, then gradually cooled to reach ambient condition. Raw samples were cut by saw and then prepared by milling. Thermal conductivity test samples were prepared on the lathe machine. SFWC radomes were made on a CNC milling machine. The surface of testing samples (tensile, compression strength test, thermal conductivity, morphology, and moisture absorption) were finished with fine-grit sandpaper to remove roughness and then cleaned.

The experimental test was performed on five specimens having a thickness of 10 mm ± 0.2 with specifications listed in Table 1. The original SFWC properties were considered as reference points in this study.

### 2.2. Weathering and Accelerated Environmental Exposure

Virgin or unaged composite samples were represented by SFWC_v_. To assess the impact of environmental aging on composite properties, test specimens were exposed to one year of weathering (real time). Conditions of accelerated environmental aging (equivalent to 20 yrs) were derived from the Hallberg peck model [22], where the acceleration factor (*AF*) was calculated by using Equation (1).

(1) $$AF=({\mathrm{RH}}_{\mathrm{stress}}-{\mathrm{RH}}_{\mathrm{use}})3\times exp[Ea/K(\frac{1}{Tuse}-\frac{1}{Tstress})]$$

Sample identifications schemes were represented as follows:

(i) virgin (sample code: SFWC_v_).
(ii) one year of weathering (sample code: A1 to A12) 1–12 months.
(iii) 20 yrs of accelerated aging (samples code: SFWC-B1 (5 yrs of aging), SFWC-B2 (10 yrs of aging), SFWC-B3 (15 yrs of aging), and SFWC-B4 (20 yrs of aging) as mentioned in Table 1 and Table 2.

SFWC specimens (SFWC-B) were exposed to accelerated environmental conditions equivalent to 5, 10, 15, and 20 years of ambient aging [19] as specified in Table 2. The temperature of 85 °C was chosen as the polymer matrix in the composite may start to show early signs of degradation (softening/interface weakening) [8]. Also, 85 °C represents a realistic upper limit of operational below glass transition temperature [14], and the industry standards recommend 85 °C as a benchmark for simulating thermal aging, i.e., IEC 60068-2-2 (for electronics and material testing) [23].

**Figure 5:**
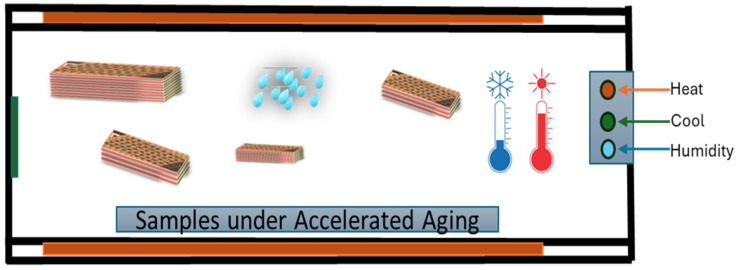
Climate chamber for accelerated aging.

SFWC-B1, SFWC-B2, SFWC-B3, and SFWC-B4 composites were exposed to accelerated environmental aging. Figure 5 represents the controlled climate chamber where the temperature and relative humidity can be reached for a specified time duration as desired.

## 3. Characterization

**Figure 6:**
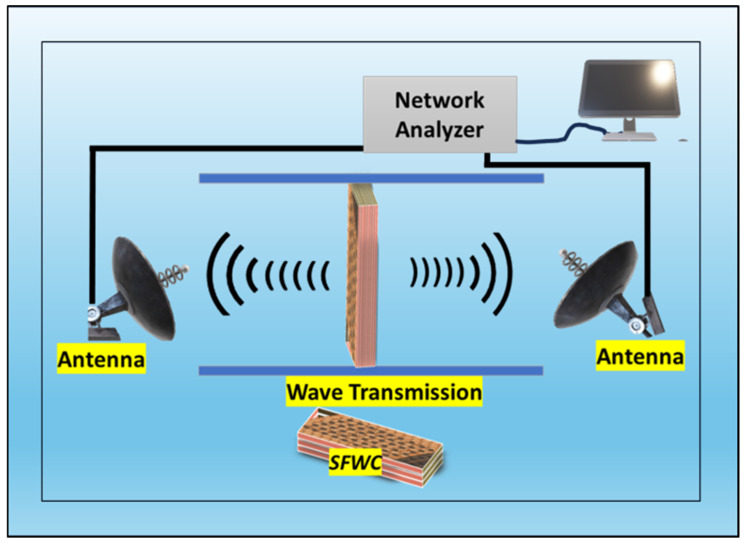
Free-space measurement set-up for dielectric properties.

At standard laboratory conditions, characterization SFWC composite physical, structural, electrical, mechanical, and thermal properties were determined. Three samples were tested for dielectric properties (ε_r_ and δ) by an impedance analyzer at X-band (8–11 GHz) using the free space method as shown in Figure 6. The morphology was determined by scanning electron microscopy (JSM-6490A, EOL, Tokyo, Japan), and chemical structure was analyzed by Fourier transform infrared (FTIR) spectroscopy.

Moisture absorption was measured (ASTM D-570 [20]) using an analytical balance (SHIMADZU 4.2). SFWC radome-enclosed antenna (with patch antenna), antenna gain, and voltage standing wave ratio (VSWR). Mechanical characterization determines material properties and behavior under applied forces. Tensile testing measures the ability of material to resist forces that attempt to pull it apart and pull it out. Compression strength quantifies the material’s ability to resist shear forces pushing inward. Tensile strength (ASTM 3039 [11]) and compression strength (ASTM 3039) were determined using the Universal Material Tester (AGX-Plus, SHIMADZU, Kyoto, Japan) at a test speed of 2 mm/min and a 50 KN load. Thermal conductivity was tested from 50 °C to 200 °C (DTC tester 300).

## 4. Results and Discussions

### 4.1. Appearance and Morphology

**Figure 7:**
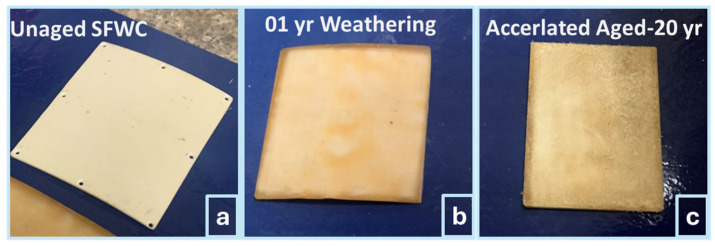
SFWC composite: (**a**) unaged, (**b**) upon weathering, and (**c**) upon accelerated aging.

**Figure 8:**
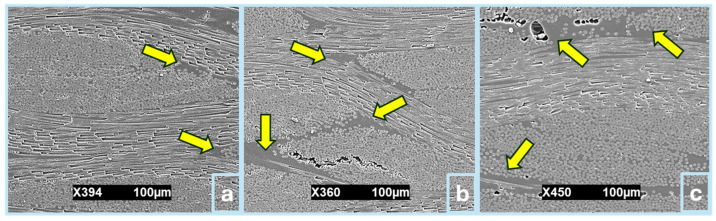
SFWC composite morphology: (**a**) unaged, (**b**) upon weathering, and (**c**) upon accelerated aging.

The physical condition and morphology of virgin/unaged (SFWC_v_), upon 1 year of weathering (SFWC-A12) and 20 yrs of accelerated aging (SFWC-B4), were shown in Figure 7 and Figure 8, respectively. The unaged SFWC_v_ radome (Figure 7a) was looking bright with a smooth surface; the one-year-aged SFWC-A12 radome (Figure 7b) was slightly whitish without a uniform texture. Environmental humidity and temperature upon weathering have brought this change in texture. SFWC-B4 radome, as shown in Figure 7c, was exposed to accelerated environmental conditions (equivalent to 20 yrs) and was observed with a rougher texture than SFWC-A12. The prolonged (hot–humid–wet environment) exposure brought change in physical appearance. The moisture diffusion occurred on the SFWC surface as it was directly exposed to a hygrothermal environment. The impact of accelerated aging can be seen from differences in their physical appearance, like further post-curing [24].

SFWC-A12’s aged surface discolored due to photo-oxidation, breakdown of resin, and reduced material glossiness as shown in Figure 7b. However, the surface of SFWC-B4, which was aged at an accelerated rate (Figure 7c), was rougher due to hot–humid conditions (moisture ingress and thermal fatigue) and led to localized surface swelling, deterioration of fiber–resin adhesion, and discoloration.

Other factors that contribute to physical appearance include fluctuations of environmental conditions, temperature, relative humidity, moisture ingress, fatigue, UV radiation (not the scope of this study), wind erosion (coastal area), and sunlight can expedite photo-oxidation of resin [25]. Despite microcracks initially present as shown in Figure 8a, SFWC_v_ fiber–matrix interactions exhibited a relatively better morphology than the aged composites. Compression at high pressure, when placed in the die-mold, where ply slippage is played, improper alignment of layers, and entanglement of fibers can result in fewer microcracks initially due to fewer air molecules being trapped. SFWC-A12 and SFWC-B4 were observed with structural differences with the initiation of fewer cracks, which are visible in Figure 8b,c.

Despite these microcracks, which can propagate upon aging, if overall composite properties (electrical, mechanical, thermal) and functional performance remain intact, then their effects can be of less consideration.

Slight discontinuities in some sections as marked in Figure 8b,c of aged composites are in agreement with reported [24] due to environmental stresses which led to weakening of interfacial bonding. In the aged composite, the stress concentration at the interface caused the beginning of more microcracks, which upon propagation can lead to declining mechanical properties or significant deterioration, as similarly studied upon long-term aggressive environments [26]. The polymer matrix in the composite becomes weakened or shows early signs of degradation due to thermal–humid–dry conditions alike [26]. Accelerated aging speeded up the moisture uptake and weakened the adhesion at the surface and fiber–matrix interface. However, there is less chance of hydrolysis occurring in silica glass fiber composites, as compared [27] to natural fiber-based composites. In accelerated aging, below the glass transition, fiber–matrix interface also becomes weakened with the initiation of debonding/slight cracks. Some portions with less compact conformation have influenced the matrix to have poor interfacial properties in agreement with the aging mechanism of E-glass fiber/epoxy composite under accelerated thermal aging [28].

### 4.2. Dielectric Constant and Dielectric Loss

**Figure 9:**
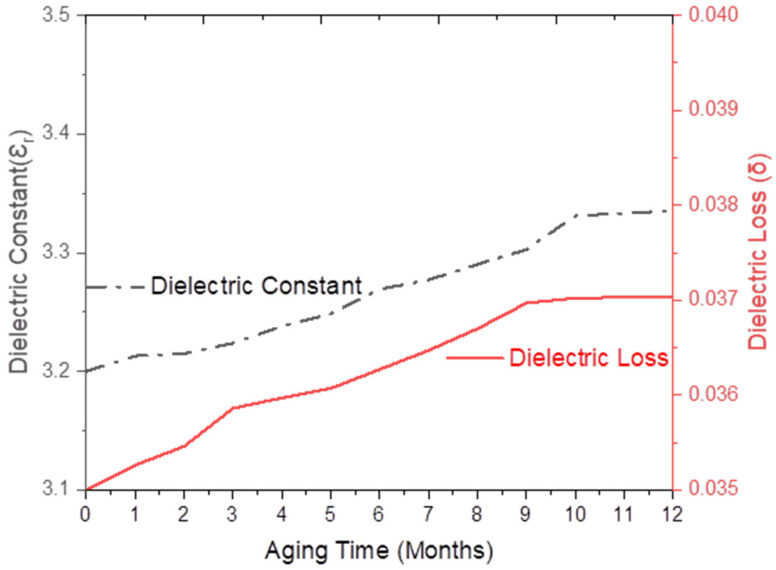
SFWC dielectric properties upon one year of aging (weathering).

Initially, the dielectric constant (ε_r_) of SFWC_v_ composite was 3.20, which, upon one year of weathering, was gradually raised to 3.33 (4.32%) as shown by Figure 9. For one yr of weathering or real aging, the composite surface was exposed to hot–humid–dry and cold weather conditions, resulting in a change in the dielectric constant due to hygrothermal exposure. The dielectric loss (δ) was 0.035, which reached 0.037 (SFWC-A12) as shown in Figure 9. Environmental (humid weather) imparted to raise this value as reported for the long-term durability study of quartz-reinforced/BMI composites [24].

**Figure 10:**
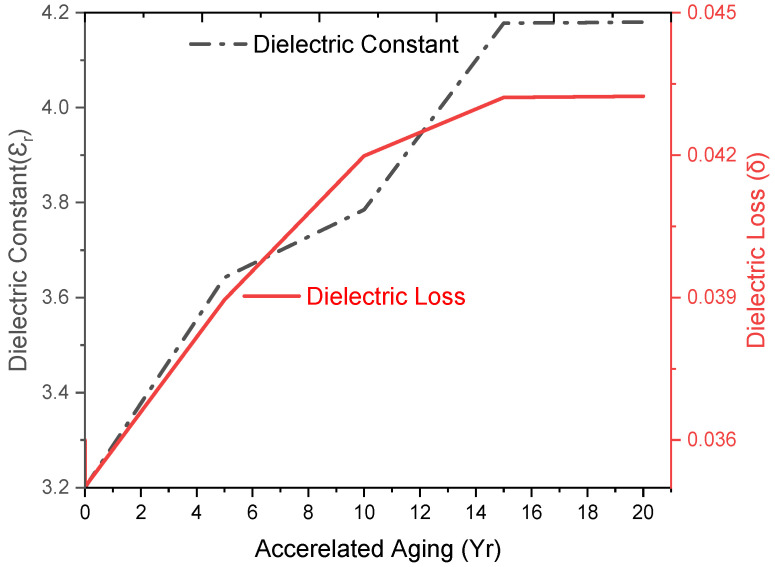
SFWC composite dielectric properties upon accelerated aging.

As shown in Figure 10, upon 5 to 20 yrs of accelerated aging, the ε_r_ was raised from 3.2 to 3.25 (5 yrs), 3.20 (10 yrs), 3.40 (15 yrs), and then retained to 4.18 (20 yrs) (30.63%). During aging from 10 to 15 years, the major increment was 30.3%, which was finally retained to 4.18 (at 20 yrs). The increasing ε_r_ was due to hot–wet–hot interaction, where hydroxyl linkage of water led to poor adhesion between the packed interfacial boundaries and increased the space charge polarization towards the electric field [29]. Accelerated aging has a higher impact than one-year weathering due to increased space charge polarization effects where moisture increased the dipole mobility and charge polarity. Increase in the δ, amplified the polarization and charge migration process, which caused a substantial transmission loss by providing a conductive effect that increased the interfacial polarization. Dielectric loss (δ) was initially 0.035, which reached 0.037 (SFWC-A12) and 0.043 (SFWC B4); an increase in the dielectric loss is related to dipole polarization at higher frequency. Overall, the accelerated aging has raised the dielectric loss to 22.8% upon 20 yrs of aging. The variation in dielectric properties is related to the environmental exposures that need enough time to reach these stringent effects [30]. Low ε_r_ and δ are desired in wave transparency applications, as δ describes how much signal is lost and delayed during its transmission. The increased δ can increase parasitic capacitance and signal delay time, which can lead to poor communication signal transmission quality.

### 4.3. Moisture Absorption

**Figure 11:**
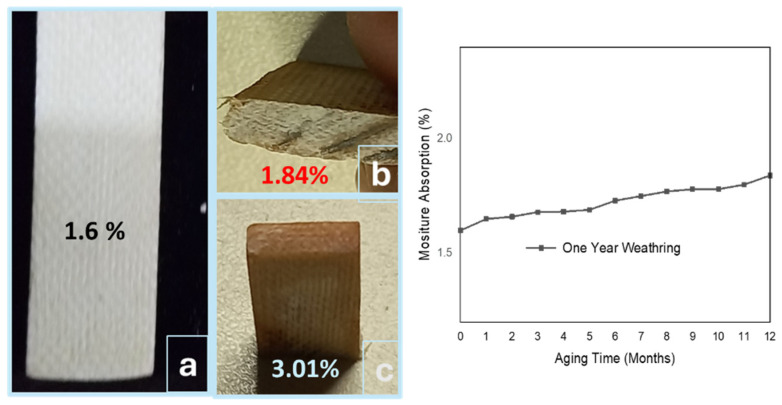
Moisture absorption: (**a**) unaged, (**b**) weathered, (**c**) and upon accelerated aging.

SFWCs are presented in Figure 11 as (a) unaged, (b) one-year aged, and (c) those with accelerated aging, where the moisture absorption was found to be 1.6% (SFWC_v_) and 1.84% (one-yr weathering). However, under accelerated conditions, moisture absorbed in SFWC-B1 (5 yrs) was 1.6%, in SFWC-B2 (10 yrs) was 2.18%, in SFWC-B3 (15 yrs) was 2.45%, and in SFWC-B4 (20 yrs) was 3.01% (the maximum). For one year of weathering in the first two months, the absorbed moisture was found on the higher side, followed by a minor increase in the last two months, as observed in Figure 11, due to changing of temperature and humidity. SFWC-B4 composite was exposed to accelerated aging conditions equivalent to 20 years and was saturated after absorbing moisture to 3.1%. Absorption of moisture can lead to performance degradation due to the higher dielectric constant (80) of water [31]. Rather than a composite compact structure, the surface is more prone to absorbing moisture. In reinforced composites, the permeation of moisture diffuses in the adhesive layer, through the interfaces, and absorption through the pores of the adherend [32]. The absorption of moisture for long durations can lead to microcrack formation, poor mechanical properties due to matrix plasticization, and stress at the interface. Moisture absorption, or water ingress, can lead to hydrolysis of the polymer matrix; weakening of fiber–resin adhesion can cause surface swelling, blistering, and expedite the microcrack propagation further (in the case of weak adhesion) and decrease the interfacial properties, resulting in fiber pull-out from the composite.

Wave-transparent composites exposure to prolonged environmental conditions can lead to poor performance and structural deterioration as well [11]. However, moisture absorption can neither be ignored nor underestimated in real scenarios; therefore, its determination in real-time weathering is critically important. The aging conditions from the Hallberg peck model [22] may deviate from the exact environmental stresses. However, the moisture absorption over the extended service life can be utilized to explore the variation analysis, which can be more effective, as it was a limited focus of electronics engineers [33].

### 4.4. SFWC Composite Radome Performance

**Figure 12:**
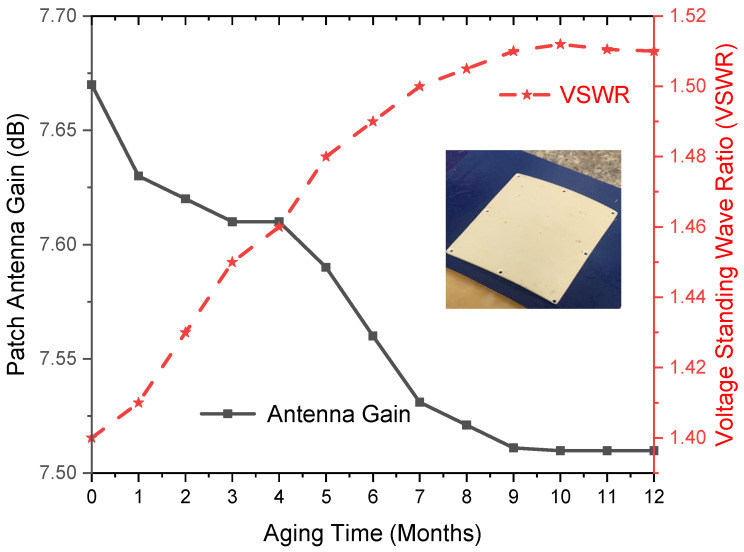
SFWC Radome Performance upon Aging.

Radomes are used to protect antennas while minimizing interference in performance [34]. Without the SFWC composite radome, the measured gain and VSWR of the patch antenna were observed to be 10.3 dB and 1.35 dB, respectively, as the radome contributes to communication signal loss [35]. The antenna is metallic and covered by a composite radome, which is non-metallic. Enclosed with the SFWC composite radome, the antenna gain reached 7.67 dB and VSWR to 1.4 dB, respectively (lowered by 2.63 dB), as shown in Figure 12. It revealed that the radiated power delivered to the antenna from its transmitter became lower, and upon aging, the increment revealed that the efficiency of the radome-enclosed antenna has been affected. It was due to an increase in ε_r_ and δ, which decreased the parasitic capacitance and signal delay time. This resulted in lower signal transmission as indicated using a multi-disciplinary approach [6].

The aging process decreased the gain and increase in VSWR due to higher dipole mobility, thus amplifying the polarization and charge migration process. This has reduced the efficiency of power transmission through radome-enclosed antennas, where more power is reflected than transmitted. High antenna gain is desired during wave transmission applications (toward and from the radome-enclosed antenna) to reduce the overall signal loss.

Antenna gain and VSWR are specified when the antenna is designed [36] (patch antenna). VSWR was below 2 (for this study), which was originally 1.4 as observed for the radome-enclosed antenna. Upon accelerated environmental aging, VSWR was raised to 1.5 (2.2% higher than original). However, this increase in VSWR is 1.5 (not an improvement factor) for a radome-enclosed antenna; nevertheless, it is reasonable for a composite radome.

SFWC radome is a reinforced composite (different from an antenna), so its characteristics may also vary as exactly specified earlier. Another reason behind this change is environmental aging, which degraded the composite properties and thus lowered the performance. These results can be implemented with consideration of other contributing factors. However, in RF communication, the VSWR below 2 is considered reasonable for the performance of radomes in ultra-wideband communication applications [35].

### 4.5. FTIR

**Figure 13:**
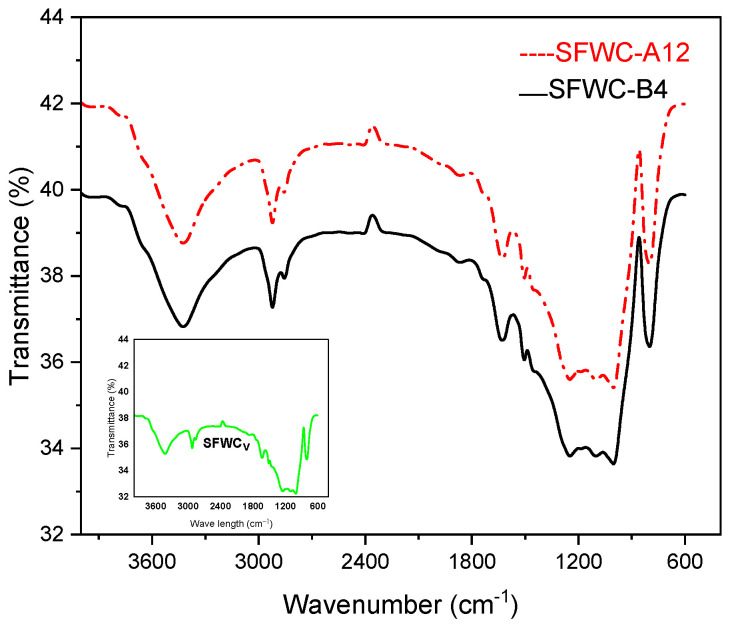
FTIR of unaged and aged SFWCs.

The FTIR spectrum is shown in Figure 13. SFWC_v_: unaged, SFWC-A12: one year of aging, and SFWC-B4: 20 yrs of aging showed –OH stretching at 3426.5 cm^−1^ and 2922.1 cm^−1^, and a peak appeared at 2855.9 cm^−1^ referred to the C-H group. The vibration peak at 1631 cm^−1^ corresponded to the C=O double bond [37]. The N-H stretching peak corresponds to 1630 cm^−1^, and an absorption peak at 1505 cm^−1^ was attributed to the N–O stretching. The peak at 1001 cm^−1^ refers to the asymmetric stretching of Si–O, and at 800 cm^−1^ it was attributed to Si–O–Si stretching vibration, and at 1246.9 cm^−1^ it referred to the C-O stretching as reported [38]. No noticeable change was observed in the basic chemical structure of the SFWC composite after weathering (one year) and accelerated environmental aging.

### 4.6. Mechanical Properties

**Figure 14:**
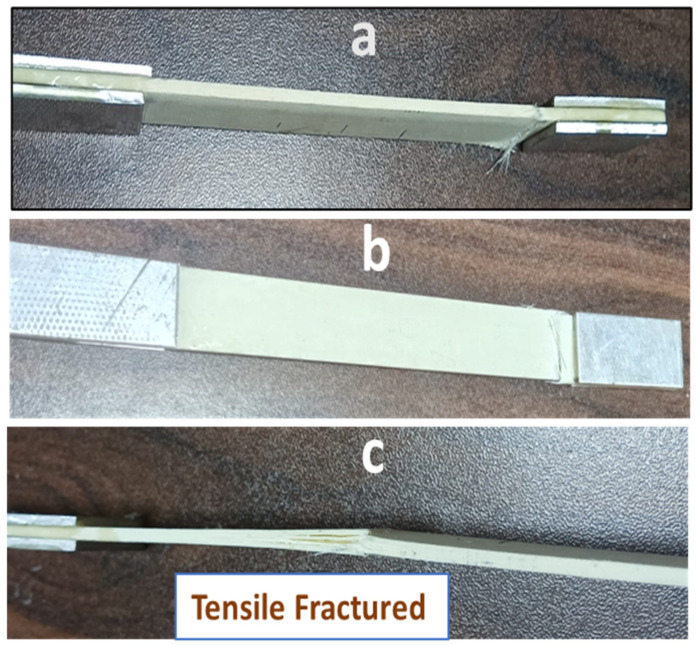
Tensile strength specimens: (**a**) unaged, (**b**) one year of aging, and (**c**) 20 yrs of aging.

**Figure 15:**
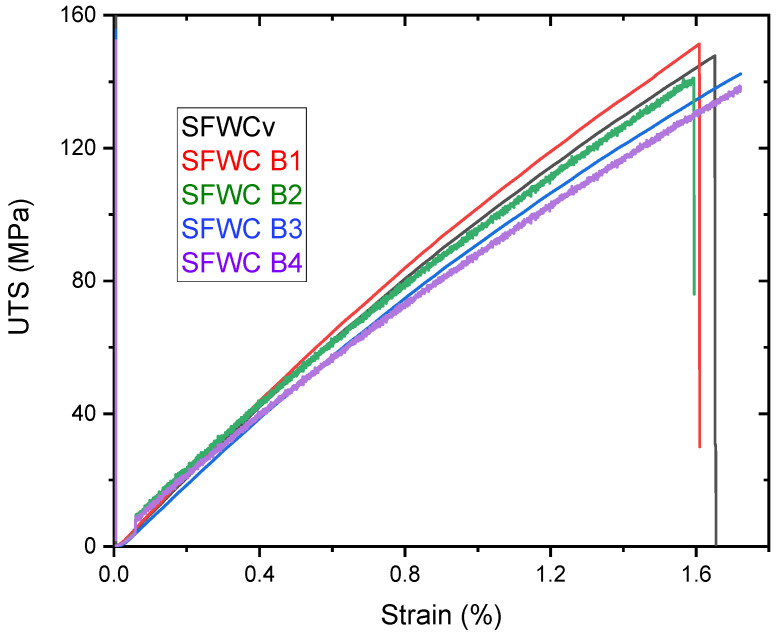
Stress-Strain During Tensile Test of SFWC composites.

Figure 14a–c shows the tensile fractured specimen, and Figure 15 represents tensile and compression strengths of the aged composites. Initially, the UTS value was 147.8 MPa, but slightly raised up to 151.3 MPa (5 yrs of aging), then decreased to 144.3 MPa (10 yrs of aging), decreased furthermore to 140.2 MPa (15 yrs of aging), and finally reached 136.48 MPa (20 yrs of aging). Initially, a slight increase in the tensile strength was supposed to be due to the post-curing effect, which was seen [39] and finally lowered upon aging. Unaged and one-year-aged samples were fractured somewhat alike, as shown in Figure 14a,b. However, the 20-year-aged sample was fractured from the midpoint with slight delamination of layers, like the hard composite fracture due to aging [21].

The value of original UCS was 388.54 MPa, which increased to 0.7% (5 yrs of aging) but ultimately declined to 374.42 MPa (4%) upon 20 yrs due to environmental aging, which deteriorated the matrix in the composite by weakening of interfacial bonding. Epoxy matrix is generally used in making wave-transparent composites. This decreasing tensile strength was due to degradation of the matrix in the cured composite because of its brittleness, which led to weak interfacial adhesion, likewise reported [40] and evident from the morphology of aged composites.

**Figure 16:**
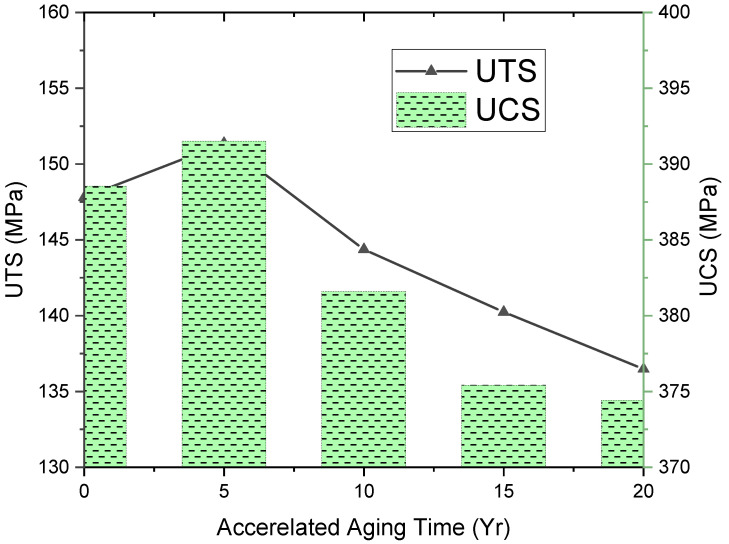
Mechanical Property Degradation of SFWC composites.

Mechanical property deterioration occurred as accelerated aging only impacted the amorphous matrix to glassy due to plasticization. Figure 15 shows the stress–strain curve obtained during the tensile test, which revealed that UTS was raised during the first 5 yrs of accelerated aging. At the start of the aging process, it seemed to be due to the post-curing effect on the SFWC composite, which increased the UTS. However, then it declined gradually until the accelerated aging conditions of up to 20 yrs. Weathering and accelerated aging both resulted in a reduction in fiber–resin bonding and mechanical performance, evident from the declined mechanical strength. As shown in Figure 16, the tensile strength reduction was slightly higher than the compression strength due to the more fiber pull-out.

### 4.7. Thermal Conductivity

**Figure 17:**
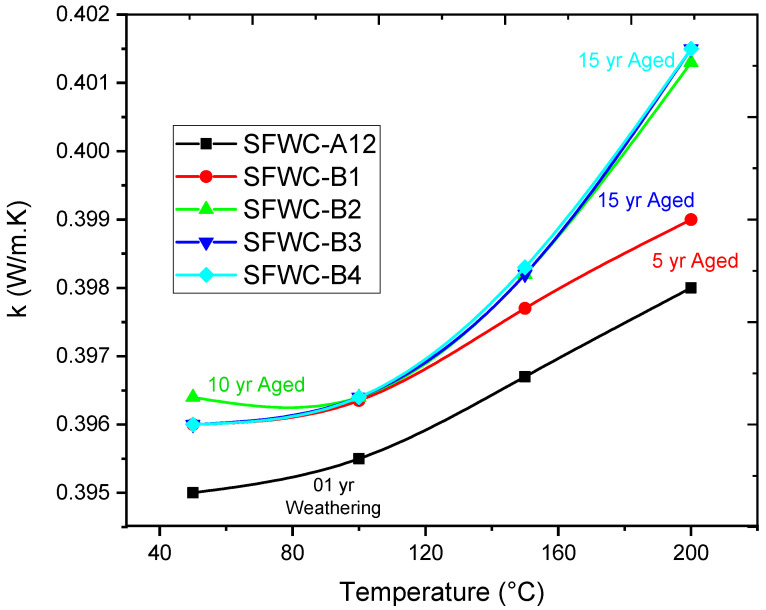
Thermal Conductivity of SFWCs.

Thermal conductivity “k” indicates how much energy a material can pass through it during [41,42]. For wave-transparent composites, the thermal conductivity is critical and can affect the transmission signals [43]. Silica fiber (HSG) and epoxy both have low thermal conductivities, and “k” of unaged SFWC was 0.398 W·m^−1^K^−1^. Upon weathering, it was 0.399 W·m^−1^K^−1^, and the accelerated aged (5, 10, 15, and 20 years) thermal conductivity values varied slightly to 0.399 W·m^−1^K^−1^, 0.401 W·m^−1^K^−1^, 0.401 W·m^−1^K^−1^, and 0.401 W·m^−1^K^−1^ (Figure 17). Measuring thermal conductivity, the rising temperature raises the “k” of epoxy more than silica fiber due to softening of resin. The moisture interacted with the hydroxyl group of the epoxy matrix and weakened the inter-chain hydrogen bonding, thus resulting in a change in the thermal conductivity value. However, in the accelerated aged composite, the amorphous devitrification has influenced the “k” of (SFWC-B3 and SFWC-B4), which was raised ~1%. Insignificant variation of thermal conductivity from its original value is attributed to the fact that the SFWC composite has a stable “k” value even upon 20 years of aging.

Overall, the thermal conductivity was slightly increased upon accelerated exposure from its original value [44]. The polymer chains in the epoxy resin were initially revealed that long-term exposure deteriorated thermosetting epoxy resin due to post-curing phenomena upon accelerated hygrothermal aging as reported [45], and thermal resistance of SFWC towards degradation was reduced from composite surfaces and interfaces.

**Figure 18:**
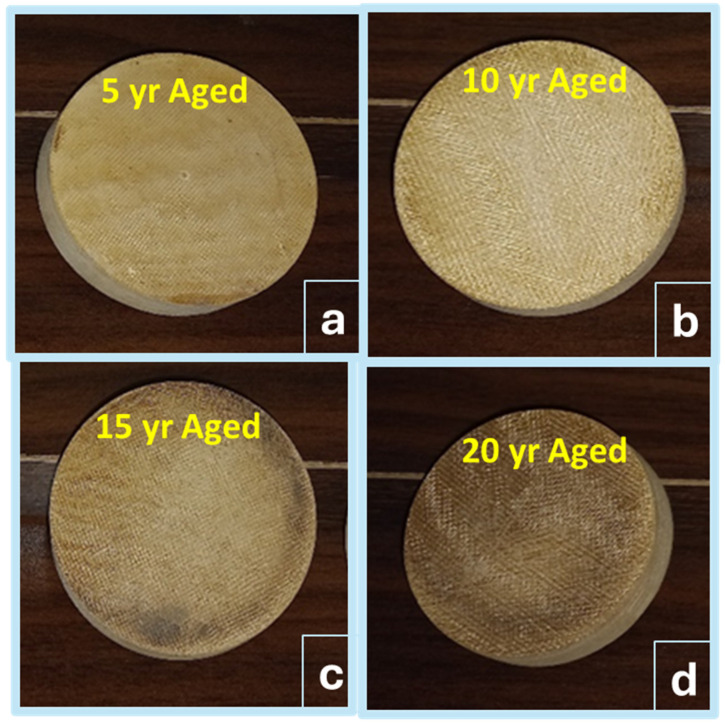
Thermal Conductivity samples (**a**–**d**) upon (5–20 yrs) accelerated aging.

At higher than ambient conditions, the resin is supposed to be post-cured, while at ambient conditions, aging phenomena happen very slowly. Aged SFWC composites as shown in Figure 18a–d where their whitish color changed to slight brown (Figure 18 (a) 5 yrs, (b) 10 yrs, (c) 15 yrs and (d) 10 yrs aged) with an apparently rough surface.

Considering the real applications as radomes (for microwave towers) are being installed from general to remote locations, expected to perform efficiently under ambient to severe environments, bear various and variable stresses (aging itself, temperature, humidity, moisture ingress, UV attack, thermal degradation, mechanical stresses, wind speed, dust, contamination, corrosive, accelerated stress, and a combination of these). Rather than studying variation of manufacturing process, this work was primarily focused on property and functional performance retention upon aging. The SFWC composite has been tested (with a patch antenna) upon long-term environmental exposure where its physical, morphological, and dielectric properties were declined but not harshly. Research is a never-ending process; however, these findings (for similar systems) can be utilized to estimate/quantify the deterioration of composite properties over a short period of time.

## 5. Conclusions

Silica fiber-reinforced wave-transparent composites (SFWCs) were investigated upon one-year ambient environmental aging and equivalent to 20 yrs of aging (via accelerated conditions) derived from the Hallberg peck model. The surface texture of the unaged SFWC composite was smooth and bright, but the aged SFWCs were rough and discolored. The morphology of SFWCs was degraded due to ambient and accelerated environmental stresses, which enhanced the weakening of interfacial bonding between fibers and matrix at composite interfaces. For one-year-aged SFWC, the dielectric constant (ε_r_) was increased to 3.75% and dielectric loss (δ) to 5.71%, whereas upon 20 yrs of accelerated aging, the ε_r_ was significantly raised 30.63% (from 3.21) and δ to 23.4% (from 0.035). Moisture absorption upon weathering was found to be 1.84%, whereas the maximum amount of moisture absorption (3.1%) was observed upon accelerated environmental hygrothermal aging (20 yrs). Not significant, but a reduction (3%) in SFWC radome-enclosed antenna was observed by of antenna gain from 7.67 to 7.5 and a small increment in VSWR to 1.4 from 1.35. Even upon aging, the identical FTIR spectra showed basic functional groups without noticeable change in SFWC structural nature. Upon 20 yrs of accelerated aging, the tensile strength and compressive strength dropped to 7% and 3.8% from their original values; however, the thermal conductivity was slightly increased (1%) from its original value. Results of this work quantified the good SFWC property retention when exposed to weathering and accelerated environmental exposure. Combining with more environmental factors can be utilized for similar glass fiber composite radome applications and would be beneficial in determining service life and mitigating earlier failures.

## Figures and Tables

**Table 1:** Real-time recorded weather conditions.

Sample Code	Aging Time	Temperature	RH
	Month	°C	%
SFWC_v_	Unaged	19.7	48
SFWC-A1	Jan	19.7	48
SFWC-A2	Feb	21.6	50
SFWC-A3	Mar	25.3	49
SFWC-A4	Apr	29.9	57
SFWC-A5	May	37.4	58
SFWC-A6	Jun	40.8	63
SFWC-A7	Jul	38.3	72
SFWC-A8	Aug	36.1	77
SFWC-A9	Sep	30.2	69
SFWC-A10	Oct	28.3	60
SFWC-A11	Nov	24.3	55
SFWC-A12	Dec	19.1	43
Total Aging Time		One-Year Weathering	

**Table 2:** SFWC-B upon accelerated weathering conditions.

Sample Code	Aging Time	Temperature	RH
	Hours	°C	%
SFWC-B1	60	85	85
SFWC-B2	80	85	85
SFWC-B3	100	85	85
SFWC-B4	120	85	85

## Data Availability

The original contributions presented in this study are included in the article. Further inquiries can be directed to the corresponding authors.
